# Using the nucleotide substitution rate matrix to detect horizontal gene transfer

**DOI:** 10.1186/1471-2105-7-476

**Published:** 2006-10-26

**Authors:** Micah Hamady, M D Betterton, Rob Knight

**Affiliations:** 1 Department of Computer Science, University of Colorado, Boulder, CO 80309, USA; 2 Department of Physics, 390 UCB, University of Colorado, Boulder, CO 80309, USA; 3 Department of Chemistry and Biochemistry, University of Colorado, Boulder, CO 80309, USA

## Abstract

**Background:**

Horizontal gene transfer (HGT) has allowed bacteria to evolve many new capabilities. Because transferred genes perform many medically important functions, such as conferring antibiotic resistance, improved detection of horizontally transferred genes from sequence data would be an important advance. Existing sequence-based methods for detecting HGT focus on changes in nucleotide composition or on differences between gene and genome phylogenies; these methods have high error rates.

**Results:**

First, we introduce a new class of methods for detecting HGT based on the changes in nucleotide substitution rates that occur when a gene is transferred to a new organism. Our new methods discriminate simulated HGT events with an error rate up to 10 times lower than does GC content. Use of models that are not time-reversible is crucial for detecting HGT. Second, we show that using combinations of multiple predictors of HGT offers substantial improvements over using any single predictor, yielding as much as a factor of 18 improvement in performance (a maximum reduction in error rate from 38% to about 3%). Multiple predictors were combined by using the random forests machine learning algorithm to identify optimal classifiers that separate HGT from non-HGT trees.

**Conclusion:**

The new class of HGT-detection methods introduced here combines advantages of phylogenetic and compositional HGT-detection techniques. These new techniques offer order-of-magnitude improvements over compositional methods because they are better able to discriminate HGT from non-HGT trees under a wide range of simulated conditions. We also found that combining multiple measures of HGT is essential for detecting a wide range of HGT events. These novel indicators of horizontal transfer will be widely useful in detecting HGT events linked to the evolution of important bacterial traits, such as antibiotic resistance and pathogenicity.

## Background

Horizontal gene transfer (HGT) has distributed genes that are required for pathogenicity among many bacterial lineages [1-4]. HGT, also known as lateral gene transfer, occurs when genes move between different genomes. Transfers can occur both between closely and distantly related species or strains, and are thought to be frequent events. For example, marine bacteriophages alone have been estimated to cause 2 × 10^16 ^horizontal transfer events per second [5]. Most of these horizontally transferred genes are lost from the population through drift or selection. However, under extreme selective pressure, such as exposure to antibiotics, some HGT genes can be stably incorporated into the genome [6-8]. In addition to genes that confer drug resistance, genes that are directly involved in pathogenicity, such as Type III secretion systems [9], have repeatedly undergone HGT [10-12]. Thus, detecting HGT has enormous practical significance for identifying new drug targets and for providing a better understanding of many bacterial pathogens.

Although HGT is thought to be an important process, the agreement between existing sequence-based HGT detection methods is surprisingly poor [13]. Estimates of the fraction of genes in modern bacterial genomes that show evidence of horizontal transfer (as opposed to vertical descent) average 5–10% across species[14-17], but can reach much higher levels. For example, initial estimates based on GC content suggested that as many as 24% of the genes in the thermophile *Thermotoga maritima *had been horizontally transferred [18]. However, more recent estimates that use different methods on the same genome place this number at 7.8%[16] and 2.0%[17]. The sensitivity of different methods for detecting HGT may correlate with the time since the transfer event [19]. These large discrepancies between methods suggest that a combination of approaches may be needed to accurately detect the full range of observable HGT events [20].

The two most popular strategies for detecting HGT from sequence data ("sequence-based methods") are phylogenetic methods and compositional methods. Both strategies have been successfully used to detect HGT events that have later been well-supported by many independent lines of evidence [21]. However, both strategies also have substantial drawbacks.

Phylogenetic methods typically compare the evolutionary history of each gene to the best estimate of the evolutionary history of the genome. For example, genes that cluster specifically with other genes from a more distantly related species, rather than with genes from a closely related species, are often inferred to have been horizontally transferred. This method has been used to identify the transfer of many genes in high-and low-temperature [18,22-24] and hospital environments [25-27]. However, many phenomena other than HGT can cause the phylogenetic tree for a gene to differ from that for the species. Thus, except in certain well-established cases of transfer of highly conserved genes such as aminoacyl-tRNA synthetases [28], phylogenetic disagreement by itself is often inconclusive [29-31]. Moreover, phylogenetic methods are also subject to false negative results, for example when there are too few closely related sequences from which to build a tree. This is particularly a problem when sequences were transferred from species that were not included in the analysis.

Compositional methods test genes for nucleotide, codon, oligonucleotide or amino acid usage that is atypical for the genome in which they are found; less typical genes may have been horizontally transferred. These methods work because genomes vary widely in GC content [32], dinucleotide frequency [33,34], and frequencies of longer oligonucleotides [35]. Therefore, genes transferred from one genome to another often differ in these properties. Compositional methods have been successful in both Gram-negative and Gram-positive bacteria. For example, compositional methods were used to identify genomic regions known as the pathogenicity islands SPI1 and SPI2 in *Salmonella enterica *[36,10], and genomic islands in *Bacillus cereus *and *B. anthracis *[37]. That compositional methods work at all is strong evidence that many HGT genes do not have the same composition as their new genomes. However, the main drawback of compositional methods is that they likely detect only recent transfer events from genomes of very different composition. This limitation arises because the composition of transferred genes changes to match the composition of the new genome [38,39].

**Figure 1:**
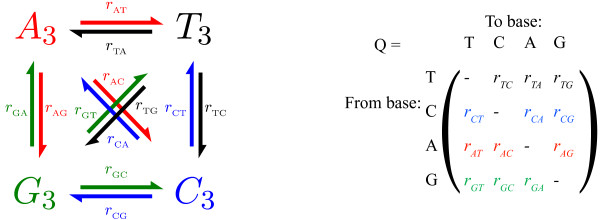
The nucleotide substitution rate matrix summarizes the instantaneous rate of change from each of the four nucleotides to each of the other four nucleotides. Each row of the rate matrix must sum to zero, and only the diagonal elements can be negative; and therefore each diagonal element is equal in magnitude but opposite in sign to the sum of the other three elements in its row. Presentation of the rates in this way is due to N. Sueoka (pers. comm.) [52, 53]. As is the convention, we estimate the rate matrix is using only the bases at the third codon position, which approximate neutral evolution. Thus, *A*_3 _refers to *A *at the third codon position, and so forth.

The limitations of these methods suggest that sequence-based HGT detection has both high false-positive and false-negative rates: many HGT events likely remain undiscovered, and many vertically inherited genes may have been misidentified as horizontally transferred. Here we present a new class of methods that detect HGT by looking for genes that evolve according to a different nucleotide substitution rate matrix than does the genome as a whole. The rate matrix consists of model parameters in the theory of neutral sequence evolution [40,41] (Figure 1) and is explained in more detail below.

Our hypothesis is that HGT changes nucleotide substitution dynamics because mutational processes differ between the old and new organisms. Thus, methods that detect changes in the rate matrix should be able to detect HGT events. We extend previous methods to detect changes in the micleotide substitution rate matrix [42,43] by (*i*) comparing three genes instead of two genes and (*ii*) studying multiple statistics derived from a set of nucleotide substitution rate matrices. To test whether this method can detect horizontally transferred genes, we simulated sequence evolution and HGT events under a wide range of conditions. We show that the new techniques we introduce here can significantly decrease the error rate when detecting simulated HGT events: a combination of four of our new statistics decreases the error rate in classifying HGT and non-HGT phylogenies up to 10-fold compared to examining variations in GC content. Our results suggest that sequence-based HGT-detection methods can be made significantly more accurate by using our new methods.

### Theory

#### Using changes in the nucleotide substitution rate matrix to detect HGT

Different species have characteristic nucleotide compositions [44], which means that they must have different rate matrices. Many mechanisms are known to affect the rate matrix. These mechanisms include loss, gain, or alteration of enzymes that affect DNA repair and/or replication, as well as production or deactivation of free radicals or other mutagens [45,46]. For example, mutations of *mutT *in *E. coli*, which encodes a DNA repair enzyme, were experimentally shown to induce directional mutation from AT to CG pairs [47], a signature of change in the rate matrix. Change in composition, and hence in the rate matrix, can be rapid on an evolutionary time scale, and large differences in composition can be observed even within a genus. For example, completely sequenced *Mycoplasma *genomes range from 24% GC in *M. mycoides *to 41% GC in *M. pneumonae *(data from CUTG [48]).

A horizontally transferred gene would typically experience an abrupt change in its rate matrix when it moves from one species to another. In contrast, all genes in a genome that have not undergone HGT should share approximately the same rate matrix. Thus, we can identify putative HGT genes as those with rate matrices that differ from the rate matrices of other genes in the same species. Different genes within a species can show small deviations in their rate matrices due to differences in transcription and replication [45]. For example, deamination proceeds much more rapidly in single-stranded DNA, so genes coded on the leading and lagging strands can experience different rate matrices [46]. In addition, differences in the rate matrix inferred from sequence data can occur due to sampling errors. However, these deviations are small compared to the differences between species.

#### The Markov model of neutral sequence evolution

The Markov model of neutral sequence evolution is a useful approximation that underlies many standard bioinformatics algorithms. These algorithms range from sequence search [49] to alignment [50] to phylogeny [51]. The model, when applied to DNA, represents the four possible nucleotides at a given position in the DNA sequence as four states T, C, A, and G. Each nucleotide has a rate of change to each other nucleotide. For example, we denote the rate of change from G to C as *r*_*G*→*C*_. These rates of change are grouped into rows and columns in the nucleotide substitution rate matrix, conventionally called *Q *(Figure 1). The rows of *Q *must sum to zero because the rate of change away from each state must balance the rate of change towards it. Thus, *Q *has negative diagonal elements and non-negative off-diagonal elements. We denote the probability of finding a G as *p*_*G *_(and similarly denote the probability of finding an A, C, and T as *p*_*A*_, *p*_*C*_, and *p*_*T*_). The model assumes *p*_*G *_changes in time according to the following equation [52,53]:

dpGdt
 MathType@MTEF@5@5@+=feaafiart1ev1aaatCvAUfKttLearuWrP9MDH5MBPbIqV92AaeXatLxBI9gBaebbnrfifHhDYfgasaacH8akY=wiFfYdH8Gipec8Eeeu0xXdbba9frFj0=OqFfea0dXdd9vqai=hGuQ8kuc9pgc9s8qqaq=dirpe0xb9q8qiLsFr0=vr0=vr0dc8meaabaqaciaacaGaaeqabaqabeGadaaakeaadaWcaaqaaiabdsgaKjabdchaWjabdEeahbqaaiabdsgaKjabdsha0baaaaa@334F@ = -(*r*_*G*→*A *_+ *r*_*G*→*C *_+ *r*_*G*→*T*_)*p*_*G *_+ *r*_*A*→*G *_*p*_*A *_+ *r*_*C*→*G *_*p*_*C *_+ *r*_*T*→*G *_*p*_*T*_.     (1)

If the probabilities of change from each single nucleotide to each other single nucleotide (e.g. T to C, T to A, T to G, and the probability that T remains in the same state) are grouped into a row vector **u**, we can write the model as dudt
 MathType@MTEF@5@5@+=feaafiart1ev1aaatCvAUfKttLearuWrP9MDH5MBPbIqV92AaeXatLxBI9gBaebbnrfifHhDYfgasaacH8akY=wiFfYdH8Gipec8Eeeu0xXdbba9frFj0=OqFfea0dXdd9vqai=hGuQ8kuc9pgc9s8qqaq=dirpe0xb9q8qiLsFr0=vr0=vr0dc8meaabaqaciaacaGaaeqabaqabeGadaaakeaadaWcaaqaaiabdsgaKHqabiab=vha1bqaaiabdsgaKjabdsha0baaaaa@3248@ = **u***Q*. These differential equations allow us to calculate the probability of change between each pair of bases after a specified amount of time *t*, because they have a formal solution for *t *≥ 0:

**u**(*t*) = **u**(0)*e*^*Qt *^= **u**(0)*P*.     (2)

The probability matrices *P*_*i *_generated from a single *Q *form a Markov semigroup with generator *Q *[54], which provides a structure that we can use to test whether a set of *P*_*i *_could have come from the same *Q*. Given a valid rate matrix *Q*, in which there are no negative off-diagonal elements, *P *= *e*^*Qt *^is guaranteed to be a probability matrix in which each row sums to 1 [54]. The probability matrix can be estimated from sequences arranged in a phylogenetic tree. Thus, *Qt *can be estimated by taking the logarithm of an empirically derived P^
 MathType@MTEF@5@5@+=feaafiart1ev1aaatCvAUfKttLearuWrP9MDH5MBPbIqV92AaeXatLxBI9gBaebbnrfifHhDYfgasaacH8akY=wiFfYdH8Gipec8Eeeu0xXdbba9frFj0=OqFfea0dXdd9vqai=hGuQ8kuc9pgc9s8qqaq=dirpe0xb9q8qiLsFr0=vr0=vr0dc8meaabaqaciaacaGaaeqabaqabeGadaaakeaacuWGqbaugaqcaaaa@2DE5@. If P^
 MathType@MTEF@5@5@+=feaafiart1ev1aaatCvAUfKttLearuWrP9MDH5MBPbIqV92AaeXatLxBI9gBaebbnrfifHhDYfgasaacH8akY=wiFfYdH8Gipec8Eeeu0xXdbba9frFj0=OqFfea0dXdd9vqai=hGuQ8kuc9pgc9s8qqaq=dirpe0xb9q8qiLsFr0=vr0=vr0dc8meaabaqaciaacaGaaeqabaqabeGadaaakeaacuWGqbaugaqcaaaa@2DE5@ is a probability matrix, then ln P^
 MathType@MTEF@5@5@+=feaafiart1ev1aaatCvAUfKttLearuWrP9MDH5MBPbIqV92AaeXatLxBI9gBaebbnrfifHhDYfgasaacH8akY=wiFfYdH8Gipec8Eeeu0xXdbba9frFj0=OqFfea0dXdd9vqai=hGuQ8kuc9pgc9s8qqaq=dirpe0xb9q8qiLsFr0=vr0=vr0dc8meaabaqaciaacaGaaeqabaqabeGadaaakeaacuWGqbaugaqcaaaa@2DE5@ = Q^
 MathType@MTEF@5@5@+=feaafiart1ev1aaatCvAUfKttLearuWrP9MDH5MBPbIqV92AaeXatLxBI9gBaebbnrfifHhDYfgasaacH8akY=wiFfYdH8Gipec8Eeeu0xXdbba9frFj0=OqFfea0dXdd9vqai=hGuQ8kuc9pgc9s8qqaq=dirpe0xb9q8qiLsFr0=vr0=vr0dc8meaabaqaciaacaGaaeqabaqabeGadaaakeaacuWGrbqugaqcaaaa@2DE7@ is guaranteed to be a *pseudo-rate *matrix with row sums equal to zero and diagonal elements each less than or equal to zero [55]. However, Q^
 MathType@MTEF@5@5@+=feaafiart1ev1aaatCvAUfKttLearuWrP9MDH5MBPbIqV92AaeXatLxBI9gBaebbnrfifHhDYfgasaacH8akY=wiFfYdH8Gipec8Eeeu0xXdbba9frFj0=OqFfea0dXdd9vqai=hGuQ8kuc9pgc9s8qqaq=dirpe0xb9q8qiLsFr0=vr0=vr0dc8meaabaqaciaacaGaaeqabaqabeGadaaakeaacuWGrbqugaqcaaaa@2DE7@ may also have negative off-diagonal elements, making it an invalid rate matrix.

Any sequence that mutates according to this model under a specified *Q *will eventually reach a steady-state composition, a process called equilibration or amelioration [38]. The equilibirum composition is given by the eigenvector of *Q *corresponding to the zero eigenvalue. Thus, the model can be used to predict the extent of equilibration of a transferred sequence to its new genome composition after a specified time. Because each *Q *has a characteristic steady-state composition, sequences with different compositions must either be under selection or differ in *Q*.

Although widely used and generally useful, the Markov model requires some assumptions that are not always biologically justified. For example, this model assumes all sites are identical and that sites are uncorrelated with each other. However, sites are often correlated in DNA sequences, especially those that encode RNA [56]. Most previous work also assumes that the mutational process is constant in time and time-reversible [57,58], but these assumptions are frequently violated by biological sequences [59]. In particular, methods for phylogenetic inference typically constrain *Q *to reduce the number of free parameters [60]. However, these constraints limit our ability to infer the true form of the matrix [61,62]. Although some of these problems, such as correlations between sites, affect all methods that use Markov models, we were able to decrease the error rate by not assuming a time-reversible Markov process. Comparisons between pairs of modern sequences force a time-reversible model because the ancestral state is not known. Consequently, it is impossible to determine whether the change was from the state in the first sequence to the state in the second sequence, or vice versa. Our use of rooted triples (rather than pairs) of sequences both allows more accurate inference of *Q *and allows the direction of each change to be inferred, which is useful when *Q *is not time-reversible.

We also limit the influence of selection on our results by using only nucleotides at the third codon position, at which changes are typically not under selection [63].

#### Mathematics of detecting changes in Q

We hypothesize that HGT causes abrupt changes in the rate matrix experienced by a gene. We therefore scan the sequences of genes within an individual genome, looking for abrupt changes in *Q*. If changes in *Q *occur during the process of evolution of a set of homologous genes (e.g. because of an HGT event), a model that assumes a single *Q *should fit the data poorly. For example, suppose that we compare each triple of homologous genes in a phylogenetic tree, and use the differences to infer a set of probability matrices P^
 MathType@MTEF@5@5@+=feaafiart1ev1aaatCvAUfKttLearuWrP9MDH5MBPbIqV92AaeXatLxBI9gBaebbnrfifHhDYfgasaacH8akY=wiFfYdH8Gipec8Eeeu0xXdbba9frFj0=OqFfea0dXdd9vqai=hGuQ8kuc9pgc9s8qqaq=dirpe0xb9q8qiLsFr0=vr0=vr0dc8meaabaqaciaacaGaaeqabaqabeGadaaakeaacuWGqbaugaqcaaaa@2DE5@_*i *_for the homologous group. We then infer the rate matrices using Q^
 MathType@MTEF@5@5@+=feaafiart1ev1aaatCvAUfKttLearuWrP9MDH5MBPbIqV92AaeXatLxBI9gBaebbnrfifHhDYfgasaacH8akY=wiFfYdH8Gipec8Eeeu0xXdbba9frFj0=OqFfea0dXdd9vqai=hGuQ8kuc9pgc9s8qqaq=dirpe0xb9q8qiLsFr0=vr0=vr0dc8meaabaqaciaacaGaaeqabaqabeGadaaakeaacuWGrbqugaqcaaaa@2DE7@*t*_*i *_= ln P^
 MathType@MTEF@5@5@+=feaafiart1ev1aaatCvAUfKttLearuWrP9MDH5MBPbIqV92AaeXatLxBI9gBaebbnrfifHhDYfgasaacH8akY=wiFfYdH8Gipec8Eeeu0xXdbba9frFj0=OqFfea0dXdd9vqai=hGuQ8kuc9pgc9s8qqaq=dirpe0xb9q8qiLsFr0=vr0=vr0dc8meaabaqaciaacaGaaeqabaqabeGadaaakeaacuWGqbaugaqcaaaa@2DE5@_*i*_. Suppose all the genes in the homologous group evolved under a single *Q*. Other than noise, the only differences between the different Q^
 MathType@MTEF@5@5@+=feaafiart1ev1aaatCvAUfKttLearuWrP9MDH5MBPbIqV92AaeXatLxBI9gBaebbnrfifHhDYfgasaacH8akY=wiFfYdH8Gipec8Eeeu0xXdbba9frFj0=OqFfea0dXdd9vqai=hGuQ8kuc9pgc9s8qqaq=dirpe0xb9q8qiLsFr0=vr0=vr0dc8meaabaqaciaacaGaaeqabaqabeGadaaakeaacuWGrbqugaqcaaaa@2DE7@*t*_*i *_will be that they come from probability matrices representing different amounts of time since divergence. In this case, all the inferred Q^
 MathType@MTEF@5@5@+=feaafiart1ev1aaatCvAUfKttLearuWrP9MDH5MBPbIqV92AaeXatLxBI9gBaebbnrfifHhDYfgasaacH8akY=wiFfYdH8Gipec8Eeeu0xXdbba9frFj0=OqFfea0dXdd9vqai=hGuQ8kuc9pgc9s8qqaq=dirpe0xb9q8qiLsFr0=vr0=vr0dc8meaabaqaciaacaGaaeqabaqabeGadaaakeaacuWGrbqugaqcaaaa@2DE7@*t*_*i *_are scalar multiples of one another. Therefore a singular-value decomposition (SVD) of the set of rate matrices should show that the largest singular value is sufficient to describe the set of rate matrices [42]. When many singular values are required to describe the set Q^
 MathType@MTEF@5@5@+=feaafiart1ev1aaatCvAUfKttLearuWrP9MDH5MBPbIqV92AaeXatLxBI9gBaebbnrfifHhDYfgasaacH8akY=wiFfYdH8Gipec8Eeeu0xXdbba9frFj0=OqFfea0dXdd9vqai=hGuQ8kuc9pgc9s8qqaq=dirpe0xb9q8qiLsFr0=vr0=vr0dc8meaabaqaciaacaGaaeqabaqabeGadaaakeaacuWGrbqugaqcaaaa@2DE7@*t*_*i *_the different inferred rate matrices for different groups of genes from the same phylogenetic tree are not scalar multiples of each other and the single-*Q *model fails to explain the data.

We are especially interested in detecting HGT in cases where genes in different parts of a phylogenetic tree have evolved under precisely two different *Q*, because this suggests a single transfer between different species. To detect this situation, we compare the uncentered and centered SVD of the set of inferred rate matrices from homologous genes within a single phylogenetic tree. In uncentered SVD (USVD), we perform the SVD on the raw Q^
 MathType@MTEF@5@5@+=feaafiart1ev1aaatCvAUfKttLearuWrP9MDH5MBPbIqV92AaeXatLxBI9gBaebbnrfifHhDYfgasaacH8akY=wiFfYdH8Gipec8Eeeu0xXdbba9frFj0=OqFfea0dXdd9vqai=hGuQ8kuc9pgc9s8qqaq=dirpe0xb9q8qiLsFr0=vr0=vr0dc8meaabaqaciaacaGaaeqabaqabeGadaaakeaacuWGrbqugaqcaaaa@2DE7@*t*_*i*_. This requires that the zero matrix is the origin of the space, and all basis vectors found in the SVD must pass through this origin. This coordinate system works well when all the Q^
 MathType@MTEF@5@5@+=feaafiart1ev1aaatCvAUfKttLearuWrP9MDH5MBPbIqV92AaeXatLxBI9gBaebbnrfifHhDYfgasaacH8akY=wiFfYdH8Gipec8Eeeu0xXdbba9frFj0=OqFfea0dXdd9vqai=hGuQ8kuc9pgc9s8qqaq=dirpe0xb9q8qiLsFr0=vr0=vr0dc8meaabaqaciaacaGaaeqabaqabeGadaaakeaacuWGrbqugaqcaaaa@2DE7@*t*_*i *_are related by scalar multiplication, because the zero matrix is then a part of the set. In centered SVD (CSVD), we perform the SVD on either the covariance matrix or the correlation matrix of the set of Q^
 MathType@MTEF@5@5@+=feaafiart1ev1aaatCvAUfKttLearuWrP9MDH5MBPbIqV92AaeXatLxBI9gBaebbnrfifHhDYfgasaacH8akY=wiFfYdH8Gipec8Eeeu0xXdbba9frFj0=OqFfea0dXdd9vqai=hGuQ8kuc9pgc9s8qqaq=dirpe0xb9q8qiLsFr0=vr0=vr0dc8meaabaqaciaacaGaaeqabaqabeGadaaakeaacuWGrbqugaqcaaaa@2DE7@*t*_*i*_. This technique is also called principal components analysis (PCA). CSVD has an effect similar to subtracting the mean of the Q^
 MathType@MTEF@5@5@+=feaafiart1ev1aaatCvAUfKttLearuWrP9MDH5MBPbIqV92AaeXatLxBI9gBaebbnrfifHhDYfgasaacH8akY=wiFfYdH8Gipec8Eeeu0xXdbba9frFj0=OqFfea0dXdd9vqai=hGuQ8kuc9pgc9s8qqaq=dirpe0xb9q8qiLsFr0=vr0=vr0dc8meaabaqaciaacaGaaeqabaqabeGadaaakeaacuWGrbqugaqcaaaa@2DE7@*t*_*i *_from each Q^
 MathType@MTEF@5@5@+=feaafiart1ev1aaatCvAUfKttLearuWrP9MDH5MBPbIqV92AaeXatLxBI9gBaebbnrfifHhDYfgasaacH8akY=wiFfYdH8Gipec8Eeeu0xXdbba9frFj0=OqFfea0dXdd9vqai=hGuQ8kuc9pgc9s8qqaq=dirpe0xb9q8qiLsFr0=vr0=vr0dc8meaabaqaciaacaGaaeqabaqabeGadaaakeaacuWGrbqugaqcaaaa@2DE7@*t*_*i*_, thus centering the coordinate space on the mean of the Q^
 MathType@MTEF@5@5@+=feaafiart1ev1aaatCvAUfKttLearuWrP9MDH5MBPbIqV92AaeXatLxBI9gBaebbnrfifHhDYfgasaacH8akY=wiFfYdH8Gipec8Eeeu0xXdbba9frFj0=OqFfea0dXdd9vqai=hGuQ8kuc9pgc9s8qqaq=dirpe0xb9q8qiLsFr0=vr0=vr0dc8meaabaqaciaacaGaaeqabaqabeGadaaakeaacuWGrbqugaqcaaaa@2DE7@*t*_*i*_. Using the correlation matrix instead of the covariance matrix has the additional effect of scaling each axis to the same coordinate system, controlling for different amounts of variation in different variables [64]. In CSVD, the origin of the coordinate system corresponds to the average *Q*, not the zero matrix. Therefore the axes corresponding to large singular values need not correspond to directions in which the original rate matrices are related by scalar multiplication. This coordinate system is particularly suited to detecting changes in *Q *(due to HGT, for example).

**Figure 2:**
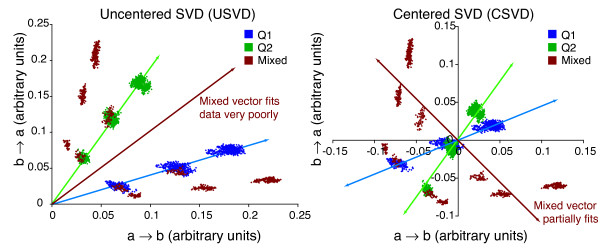
Relationship between (a) uncentered SVD and (b) centered SVD. A set of related sequences is used to determine a set of rate matrices, *Q*_*i*_. If the sequences were generated by mutation with the same rate matrix for all species throughout time, then the rate matrices are all scalar multiples of each other. In this case, the singular-value decomposition (SVD) of the set of *Q*_*i *_will show that a single vector (corresponing to the time axis) explains most of the variation in the set of rate matrices. Both USVD (the SVD is performed on the *Q*_*i *_directly) and CSVD (the SVD is performed on the covariance matrix of the *Q*_*i*_) will find the same dominant axis. If the rate matrix is different for different species or time-varying, no single vector will explain most of the variation in the set of rate matrices. In other words, USVD will not find a single best axis. However, for the case of precisely two rate matrices, CSVD, but not USVD, will still find a single, non-time axis that explains much of the variation in the rate matrices (in the example shown, *r*^2 ^= 0.46 for the best-fit line through the mixed points on the right panel). This occurs because a single vector explains much of the variance in the set of rate matrices, but this vector does not correspond to a time axis, and hence cannot be found in USVD. Data shown are for a simplified model of rate matrices with a two-character alphabet a and b instead of the four-character alphabet used in DNA. In this simplified alphabet, *Q *has only two non-negative elements representing *r*_*a*→*b *_and *r*_*b*→*a*_. These two elements are plotted on the *x *and *y *axes of each graph. Data shown are for 16-taxon trees evolving according to the single rate matrix *r*_*a*→*b *_= 0.9, *r*_*b*→*a *_= 0.1 (blue points), *r*_*a*→*b *_= 0.2, *r*_*b*→*a *_= 0.8 (green points), or an equal mixture of both (red points).

For a set of homologous sequences related by a single *Q*, USVD and CSVD should both find a good fit, because most of the variance in the rate matrices is due to variation in time, which corresponds to scalar multiplication (Figure 2). Both USVD and CSVD should also find the same axis, which accounts for most of the variation in the set of rate matrices obtained from different combinations of genes in the tree. For a set of homologous sequences evolved under two different rate matrices, USVD should fit the single-*Q *model poorly, because the variation in the inferred rate matrices, Q^
 MathType@MTEF@5@5@+=feaafiart1ev1aaatCvAUfKttLearuWrP9MDH5MBPbIqV92AaeXatLxBI9gBaebbnrfifHhDYfgasaacH8akY=wiFfYdH8Gipec8Eeeu0xXdbba9frFj0=OqFfea0dXdd9vqai=hGuQ8kuc9pgc9s8qqaq=dirpe0xb9q8qiLsFr0=vr0=vr0dc8meaabaqaciaacaGaaeqabaqabeGadaaakeaacuWGrbqugaqcaaaa@2DE7@*t*_*i*_, is influenced both by variation in *t *and by variation in *Q*. On the other hand, CSVD should find a good fit because it subtracts out the time variation: most of the variance will be explained by a single axis that links the two clusters from the two distinct *Q *(Figure 2).

We propose that comparison of USVD and CSVD fits can reveal when a single change in *Q *occurred. We expect that this method will work better for larger differences in *Q*, and where the differences are not solely due to scalar multiplication. Below, we address precisely how large a difference this method can detect using simulated sequence evolution.

## Results

### Inferring the rate matrix

**Figure 3:**
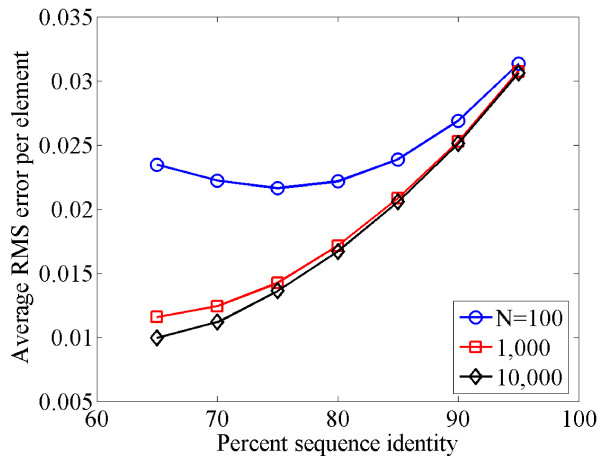
Average root mean square (RMS) error per matrix element when estimating the rate matrix *Q *from simulated sequence data. Curves are shown for three different sequence lengths, *N *= 100, 1000, and 10000. Note that the increase in the error for higher percent sequence identity is a sampling effect: more similar sequences have fewer sites of change, and therefore the P^
 MathType@MTEF@5@5@+=feaafiart1ev1aaatCvAUfKttLearuWrP9MDH5MBPbIqV92AaeXatLxBI9gBaebbnrfifHhDYfgasaacH8akY=wiFfYdH8Gipec8Eeeu0xXdbba9frFj0=OqFfea0dXdd9vqai=hGuQ8kuc9pgc9s8qqaq=dirpe0xb9q8qiLsFr0=vr0=vr0dc8meaabaqaciaacaGaaeqabaqabeGadaaakeaacuWGqbaugaqcaaaa@2DE5@ determined from the data have higher sampling error.

We first checked that we can recover the rate matrix accurately enough for inferred differences in *Q *to be valid. This is a critical first step in demonstrating that the method works. The main sources of error are sampling error from the finite amount of sequence data, and numerical error introduced by the matrix logarithm and exponentiation. Both numerical error and sampling error can lead to the inference of a pseudo-rate matrix with negative off-diagonal elements when taking the logarithm of *P*. To estimate the effects of sampling, we compared each P^
 MathType@MTEF@5@5@+=feaafiart1ev1aaatCvAUfKttLearuWrP9MDH5MBPbIqV92AaeXatLxBI9gBaebbnrfifHhDYfgasaacH8akY=wiFfYdH8Gipec8Eeeu0xXdbba9frFj0=OqFfea0dXdd9vqai=hGuQ8kuc9pgc9s8qqaq=dirpe0xb9q8qiLsFr0=vr0=vr0dc8meaabaqaciaacaGaaeqabaqabeGadaaakeaacuWGqbaugaqcaaaa@2DE5@ determined from the sequence to the expected *P *using *P *= *e*^*Q*^, and found a sampling error of 0.0075 to 0.025 (data not shown). Thus our method gives errors comparable to the intrinsic sampling error. We found that the sampling error was relatively small when the sequence length exceeded 1000 nucleotides (Figure 3), a reasonable estimate for a single gene.

### Variation in the rate matrix within and between genomes

We used both simulated sequence evolution and representative bacterial genome sequences to (*i*) test how many genes are required for accurate inference of the rate matrix and (*ii*) confirm that the rate matrices estimated from genes in a single species are more similar to each other than to rate matrices estimated from genes in different genomes. We expected that when we increased the number of genes *n *used to determine the rate matrix, the estimated rate matrices would become more accurate. For large *n *the estimated rate matrix should converge to the average rate matrix for the genome. For the rate matrix estimates from bacterial sequence data, we chose sets of three genomes (triples) at approximately the same level of divergence as the sequences used in the simulations (see Methods for details and a list of bacterial genomes used in the analysis).

**Figure 4:**
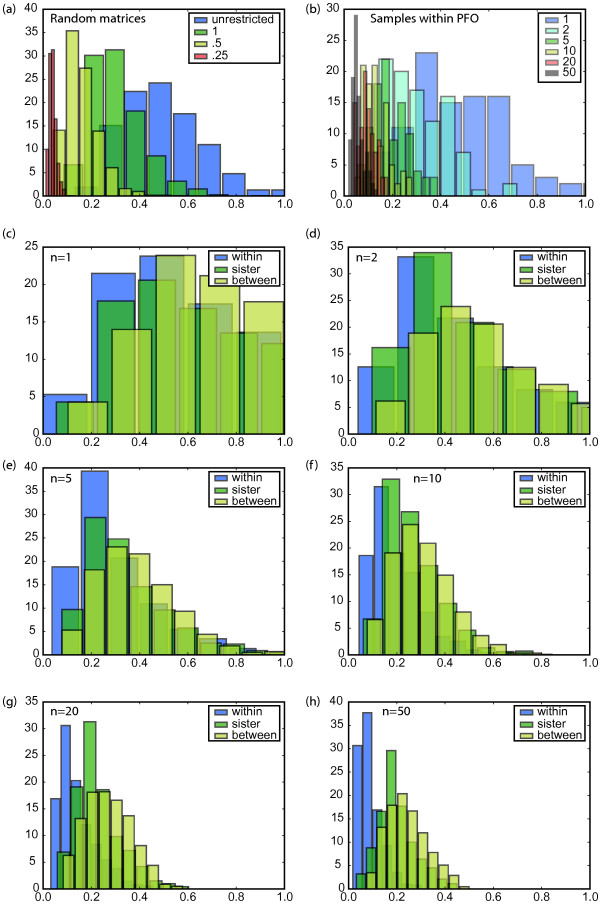
Average difference between two *Q*_*i *_obtained from samples of genes within a genome ('within'), between sister genomes ('sister'), or between more distantly related genomes ('between'). Panel (a) shows results for simulated sequence evolution. Panels (b)-(h) show results from fully sequenced bacterial genomes; see Methods for details and a list of species included, (a) Distances for pairs randomly-generated matrices, using either the unrestricted model ('unrestricted') or by perturbing a starting matrix by a specified amount (100%, 50%, or 25% per element). (b) Results from one representative genome, PFO (see Methods). Series represent number of genes *n *used to estimate the rate matrix. By the time *n *increases to 10 genes (yellow series), the within-genome distance is a factor of 2–4 smaller than any between-genome distance, (c-h) Results for comparisons averaged over all genomes. As the sample size *n *increases from 1 to 50, the separation between the within-species and between-species distribution increases. This separation is highly significant within each species by *n *= 10 (for example, for PFO, the difference is about a factor of 2; *P *< 10^-300^).

Figure 4 shows these comparisons (a) for randomly generated rate matrices at different specified levels of divergence (Figure 4a), (b) for rate matrices generated from different samples of genes in one representative genome, PFO (*Pseudomonas fluorescens Pf0-1*), which we use as an example (Figure 4b), and (c) for comparisons within and between genomes for all species triples in the comparison, where we varied the number of genes *n *used to estimate the rate matrix from *n *= 1 to *n *= 50 (Figure 4c–h). When the rate matrix is estimated based on samples of ten or more genes, the mean distance in the rate matrix between pairs of samples taken from the *same genome was always substantially less than the mean distance between pairs taken from *different genomes (assessed by two-sample *t *test: *t *= 28.6, *P *< 10^-173^). We note that when the rate matrix estimate is based on single genes, the estimated rate matrices are overdispersed (more different than would be expected for randomly chosen rate matrices). This effect occurs because small sample sizes lead to counts of zero for some changes, which can cause the rate matrices to appear radically different.

To infer how different the rate matrix is between different genomes, we used the rate matrix estimates based on samples of 50 genes (Figure 4g; see Methods for details). For samples of genes taken from the same genome, the average distance between rate matrices is only 0.09. When we compare estimated rate matrices of sister genomes, the average distance between rate matrices is 0.20, while for distantly related genomes the average distance between rate matrices is 0.25. We note that rate matrices of sister genomes look nearly as different from each other, on average, as do the rate matrices of distantly related species.

The average distances between rate matrices which we estimate from biological sequence data correspond to per-element perturbations of 0.55–0.65. This within the range of matrix divergences we used in our simulations. The genomes used for the comparison were all relatively GC-rich. Therefore we expect the rate matrices to differ substantially more for genomes with greater compositional differences. These results demonstrate that our conclusions based on simulated sequence data are likely to be relevant to biological sequences.

### Discriminating among phylogenies generated with one, two, or many rate matrices

We found that we can determine whether a phylogeny was generated by one, two, or many rate matrices with greater accuracy than allowed by previous methods. We used a total of 45 different statistics based on properties of the set of *Qt*_*i *_inferred from homologous sequences within each simulated phylogenetic tree (Table 1) to test whether we could distinguish among single-*Q*, double-*Q*, and multi-*Q *phylogenies. We found that many of the statistics offered substantial improvement over previously published statistics for detecting changes in the rate matrix.

**Table 1:** Statistics used to detect changes in the rate matrix.

#	Type	Sequences	Normalized	Centered	SV measure	Centered SVD method	Rank	%
1	SVD	2	No	Yes	a	Correlation	44	0.4
2	SVD	2	No	Yes	b	Correlation	40	0.5
3	SVD	2	No	Yes	c	Correlation	12	2.5
4	SVD	2	No	Yes	a	Covariance	41	0.5
5	SVD	2	No	Yes	b	Covariance	42	0.5
6	SVD	2	No	Yes	c	Covariance	37	0.6
7	SVD	2	No	No	a	_	36	0.6
8	SVD	2	No	No	b	_	39	0.5
9	SVD	2	No	No	c	_	35	0.8
10	Mean(d)	2	No	-	-	-	34	0.9
11	Var(d)	2	No	-	-	-	22	1.8
12	SVD	2	Yes	Yes	a	Correlation	23	1.8
13	SVD	2	Yes	Yes	b	Correlation	43	0.5
14	SVD	2	Yes	Yes	c	Correlation	32	1.2
15	SVD	2	Yes	Yes	a	Covariance	14	2.2
16	SVD	2	Yes	Yes	b	Covariance	45	0.3
17	SVD	2	Yes	Yes	c	Covariance	18	2.0
18	SVD	2	Yes	No	a	_	28	1.5
19	SVD	2	Yes	No	b	_	21	1.9
20	SVD	2	Yes	No	c	_	31	1.4
21	Mean(d)	2	Yes	-	-	-	24	1.7
22	Var(d)	2	Yes	-	-	-	33	1.2
23	SVD	3	No	Yes	a	Correlation	7	3.9
24	SVD	3	No	Yes	b	Correlation	26	1.6
25	SVD	3	No	Yes	c	Correlation	20	1.9
26	SVD	3	No	Yes	a	Covariance	6	3.9
27	SVD	3	No	Yes	b	Covariance	19	1.9
28	SVD	3	No	Yes	c	Covariance	2	6.9
29	SVD	3	No	No	a	_	8	3.8
30	SVD	3	No	No	b	_	30	1.5
31	SVD	3	No	No	c	_	13	2.3
32	Mean(d)	3	No	-	-	-	3	6.8
33	Var(d)	3	No	-	-	-	10	3.3
34	SVD	3	Yes	Yes	a	Correlation	5	5.1
35	SVD	3	Yes	Yes	b	Correlation	15	2.2
36	SVD	3	Yes	Yes	c	Correlation	4	5.7
37	SVD	3	Yes	Yes	a	Covariance	1	7.7
38	SVD	3	Yes	Yes	b	Covariance	29	1.5
39	SVD	3	Yes	Yes	c	Covariance	16	2.1
40	SVD	3	Yes	No	a	-	9	3.5
41	SVD	3	Yes	No	b	-	27	1.6
42	SVD	3	Yes	No	c	-	38	0.6
43	Mean(d)	3	Yes	-	-	-	17	2.1
44	Var(d)	3	Yes	-	-	-	25	1.7
45	Var(GC)	-	-	-	-	-	11	3.2

Table of 45 statistics derived from collections of rate matrices. Columns are: #: number of each method, used in the text for reference. Type: type of statistic, either SVD, mean distance of each *Q*_*i *_from the average of all *Q*_*i *_(Mean(d)), variance of the distances of the *Q*_*i *_from the average of all *Q*_*i *_(Var(d)), or variance in GC content (Var(GC)). Sequences: either 2, for pairwise sequence comparisons that assume a time-reversible substitution model, or 3, for three-way sequence comparisons that do not assume a time-reversible model. Normalized: either No, for unnormalized *Q*_*i*_, Yes, for *Q*_*i *_normalized to a trace of 1 (eliminating the contribution of time to the inferred matrix), or N/A, where normalization was not applicable. Centered: Yes, for CSVD, No, for USVD, or N/A, where SVD was not used. SV measure: statistic for characterizing singular values, either a, for ratio of the two largest singular values, or b, for ratio of the largest singular value to the sum of all singular values, or c, for ∑_*i *_ln(1 + *σ*_*i*_), where the *σ*_*i *_are the singular values (this is the SVD version of the statistic introduced by Weiss et al. [43]), or N/A, where not applicable. CSVD method: Covariance if the covariance matrix was used for CSVD, Correlation if the correlation matrix was used, N/A if not applicable (i.e., if the technique was not centered SVD). Rank: rank of the statistic in contributing to overall classification accuracy using random forests [75]. %: percentage contribution of the statistic to overall classification accuracy using random forests. The statistic used by Devauchelle et al. [42] corresponds to statistic 7; that used by Weiss et al. [43] corresponds to statistic 9.

**Figure 5:**
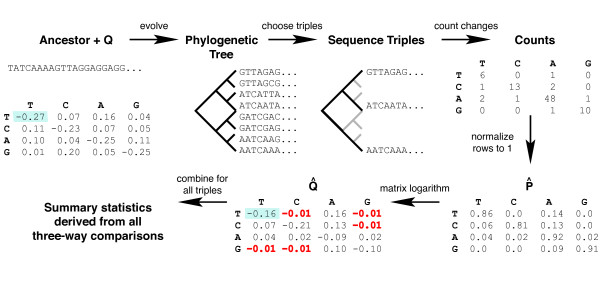
Procedure for simulating sequences. From a random ancestral sequence and one or more random rate matrices, we evolve a tree of 8 or 16 sequences. We then choose all combinations of three sequences (shown), or, for pairwise comparisons, two sequences (not shown), from the tree. For each sequence pair or triple, we infer P^
 MathType@MTEF@5@5@+=feaafiart1ev1aaatCvAUfKttLearuWrP9MDH5MBPbIqV92AaeXatLxBI9gBaebbnrfifHhDYfgasaacH8akY=wiFfYdH8Gipec8Eeeu0xXdbba9frFj0=OqFfea0dXdd9vqai=hGuQ8kuc9pgc9s8qqaq=dirpe0xb9q8qiLsFr0=vr0=vr0dc8meaabaqaciaacaGaaeqabaqabeGadaaakeaacuWGqbaugaqcaaaa@2DE5@ by counting the directed changes in each of the two sister sequences (relative to the outgroup), and infer Q^
 MathType@MTEF@5@5@+=feaafiart1ev1aaatCvAUfKttLearuWrP9MDH5MBPbIqV92AaeXatLxBI9gBaebbnrfifHhDYfgasaacH8akY=wiFfYdH8Gipec8Eeeu0xXdbba9frFj0=OqFfea0dXdd9vqai=hGuQ8kuc9pgc9s8qqaq=dirpe0xb9q8qiLsFr0=vr0=vr0dc8meaabaqaciaacaGaaeqabaqabeGadaaakeaacuWGrbqugaqcaaaa@2DE7@ by taking the log of P^
 MathType@MTEF@5@5@+=feaafiart1ev1aaatCvAUfKttLearuWrP9MDH5MBPbIqV92AaeXatLxBI9gBaebbnrfifHhDYfgasaacH8akY=wiFfYdH8Gipec8Eeeu0xXdbba9frFj0=OqFfea0dXdd9vqai=hGuQ8kuc9pgc9s8qqaq=dirpe0xb9q8qiLsFr0=vr0=vr0dc8meaabaqaciaacaGaaeqabaqabeGadaaakeaacuWGqbaugaqcaaaa@2DE5@ (after normalization to make the rows sum to 1). The inferred rate matrix Q^
 MathType@MTEF@5@5@+=feaafiart1ev1aaatCvAUfKttLearuWrP9MDH5MBPbIqV92AaeXatLxBI9gBaebbnrfifHhDYfgasaacH8akY=wiFfYdH8Gipec8Eeeu0xXdbba9frFj0=OqFfea0dXdd9vqai=hGuQ8kuc9pgc9s8qqaq=dirpe0xb9q8qiLsFr0=vr0=vr0dc8meaabaqaciaacaGaaeqabaqabeGadaaakeaacuWGrbqugaqcaaaa@2DE7@ may contain negative off-diagonal elements (shown in red); note the error in the corresponding elements of *Q *and Q^
 MathType@MTEF@5@5@+=feaafiart1ev1aaatCvAUfKttLearuWrP9MDH5MBPbIqV92AaeXatLxBI9gBaebbnrfifHhDYfgasaacH8akY=wiFfYdH8Gipec8Eeeu0xXdbba9frFj0=OqFfea0dXdd9vqai=hGuQ8kuc9pgc9s8qqaq=dirpe0xb9q8qiLsFr0=vr0=vr0dc8meaabaqaciaacaGaaeqabaqabeGadaaakeaacuWGrbqugaqcaaaa@2DE7@ (e.g. the first element highlighted in blue). Finally, we combine the *Q *from each triple and derive summary statistics.

**Figure 6:**
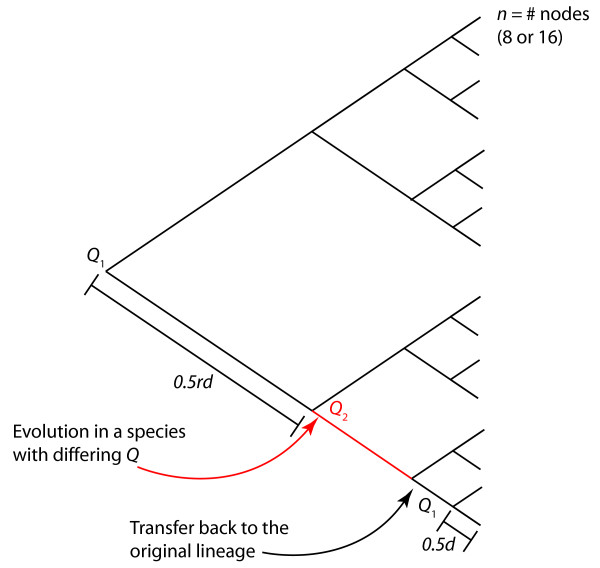
Model parameters varied during simulated evolution of phylogenies. The total number of nodes *n *corresponds to the number of sequences (taxa) at the end of the simulation. The sequences are evolved so that sister taxa have an average percent divergence *d*; the length of time from each terminal taxon to its most recent common ancestor is *d*/2. The rate matrix *Q*_1 _is fixed over the whole tree, except where the simulated horizontal transfer event occurs. The modeled HGT event occurs between the second and third internal branch points and is represented by evolving one of the four taxa according to a different rate matrix *Q*_2_. (Note that if no HGT event is modeled, *Q*_2 _= *Q*_1_). To vary how far back in time the simulated HGT event occurred, we varied the ratio *r *betweeen the first branch and all other branches. Larger *r *corresponds to a more recent HGT event. The parameters were: *n *= 8 or 16, *d *was between 0.05 and 0.5 in steps of 0.05, and *r *was 1, 2, 5, 10, or 20. The first rate matrix *Q*_1 _was chosen randomly. In the "unconstrained" case, *Q*_2_was chosen randomly, independent of *Q*_1_. In the "constrained" case, we required that each random element of *Q*_2 _be within a certain percentage of the corresponding element of *Q*_1_; percentages of 20%, 50%, and 90% were considered.

**Figure 7:**
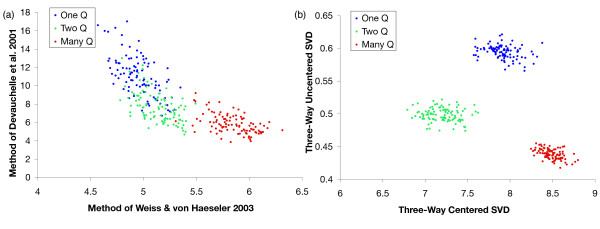
Improved discrimination between phylogenetic trees generated by one (blue), two (green) and many (red) rate matrices. (a) Previously published statistics 7 [42] and 9 [43] fail to separate single-*Q *trees from double- and multi-*Q *phylogenies. (b) Two of our new statistics, statistics 25 and 41, which distinguish single-*Q *trees efficiently. Due to sample variance, no single statistic provides adequate discrimination (i.e., the groups overlap substantially if only a single dimension is considered). However, combining two of our new statistics (see text) easily separates the single-*Q *trees from the other two groups. Data shown are from 1000 balanced 16-sequence phylogenies of each type, with average divergence of 10% between sister sequences and sequence length of 1000. These results show that we can detect changes in the rate matrix much more sensitively with our new methods than with previous methods. In these simulations, but not the other simulations shown, the same random *Q *was used for different runs of a given type of simulation.

**Figure 8:**
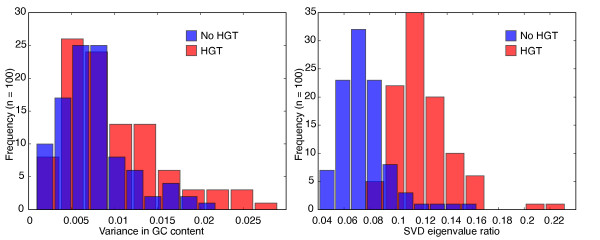
Improved detection of simulated HGT events using our new statistics. The histograms show the discrimination using GC content, statistic 45 (left), and ratio of first two singular values in three-way comparisons, statistic 41 (right). The new statistic is considerably better at classifying the HGT (red) and non-HGT (blue) phylogenies, as shown by the greater separation between the two histograms. Statistic 41 gives an optimal classifier with 1.5% false positives and 5% false negatives, a threefold improvement in discrimination over statistic 45. Data shown are from 1000 balanced 16-sequence trees of each type, with an average divergence of 10% between sister taxa.

We tested classification of three types of simulated phylogenetic trees, generated by one, two, or many rate matrices (Figure 5), according to the model shown in Figure 6. For the data shown in figures 7–8, we simulated balanced trees with 16 sequences (taxa). In the double-*Q *phylogenies, 12 of the sequences evolved according to one random *Q*, and the remaining 4 sequences evolved according to another random *Q*. In the multi-*Q *phylogenies, a new, random rate matrix was generated for each branch. Results shown are for trees with an average divergence of 10% between the most closely related sequence pairs, but results were similar for divergences ranging from 5% to 50% in steps of 5% (data not shown). Neither of the previously published statistics we tested, statistic 7 and statistic 9 [43,42], nor their use in combination, could reliably separate the three types of trees (Figure 7a). However, the combination of two of our best-performing statistics, statistic 25 and statistic 41, efficiently separated all three classes of phylogenetic trees (Figure 7b).

### Detecting simulated HGT events

Our simulation of HGT events was designed to model orthologous gene displacement. In this type of transfer event, the gene in one species is replaced by or supplemented with an ortholog from another species. (Orthologs are genes that have diverged only through speciation, as opposed to paralogs, which are genes that have diverged after duplication within a single species.) Orthologous gene displacement has been documented for several functional categories of genes, including enzymes in the citric acid cycle [65], antibiotic resistance genes such as variant dihydrofolate reductases [66], and even the genes comprising the entire type III secretion system apparatus [11]. However, these orthologous displacements are difficult to detect with existing techniques [67]. Therefore, we hypothesized a more-powerful HGT-detection method based on changes in *Q *to be especially useful for detecting orthologous gene displacement.

To design simulations of orthologous gene displacement, we assumed that genes diverge from a common ancestor. Initially, both daughter species have the same rate matrix. One of the two daughter species then undergoes a mutation that changes its rate matrix, changing the evolutionary pattern of the gene in that lineage. Finally, the gene transfers back to the other lineage (which is evolving under the original rate matrix), and undergoes a period of evolution under that original matrix. This simulated transfer event is a challenge for HGT detection methods, because orthologous gene displacement can be difficult to distinguish from loss of alternate paralogs. Furthermore, orthologous gene displacement is a challenge for methods of HGT detection that rely on nucleotide composition because of rapid equilibration to the new genome in the final period of evolution (Figure 6).

To determine which statistics could most accurately identify HGT by orthologous gene replacement, we simulated samples of balanced eight-taxon phylogenetic trees with 10% divergence between sister taxa. These trees either evolved according to a single random *Q*, or contained a horizontal transfer event (Figure 6). In the simulated HGT phylogenies, four of the sequences initially evolve under a different, random *Q*, and then transfer to taxa with the same *Q *as the remaining species. We compared the ability of our new methods, and of the standard compositional method based on variation in GC content (statistic 45), to discriminate between HGT and non-HGT phylogenies.

We focus on GC content rather than other compositional measures for two reasons. First, GC content is much more consistent within bacterial genomes than between genomes [32]. This means that variation in GC content is widely used in practice to detect genomic islands (e.g. in [11]). Second, almost all of the variation in composition in biological sequences is in GC content rather than along the other two possible axes of composition; this is true both for coding sequences [68-70] and for non-coding sequences such as rRNA [68,71,72]. The rare exceptions are in genomes that exhibit extreme bias between the leading and lagging strand [46], although these effects are small in most microbial genomes [73]. (A comprehensive comparison of compositional metrics is beyond the scope of this analysis, but we direct readers interested in these issues to recent literature on this topic [38,39,34,13,21].)

We found that our methods could detect HGT much more sensitively and specifically than by using GC content. In our simulations, the variance in GC content was not highly discriminatory (Figure 8, left). Although the difference in variance in GC content between HGT and non-HGT trees was highly significant (*P *= 7 × 10^-25 ^by unpaired 2-tailed t-test, *n *= 100 per sample), the optimal single-variable classifier [74] gave 21% false positives and 0.5% false negatives. In contrast, the best of our statistics, statistic 41, resulted in only 1.5% false positives and 5% false negatives, a three-fold improvement in overall error rate despite the increase in false negatives (Figure 8, right). The difference in this statistic was highly significant (*P *= 8 × 10^-63 ^by unpaired 2-tailed *t*-test, *n *= 100 per sample). In simulated phylogenies we can detect HGT with much higher precision than existing methods.

### Limits of accuracy

We expected that our method for HGT detection will work best when the original and new host species have very different rate matrices. To determine what changes in the rate matrix are required for accurate detection, we repeated the HGT-detection analysis with restrictions on the average variation in *Q*. This analysis indicates how recently a transfer must have occurred, and how different *Q *must have been in the organism that the gene came from, to be detectable. We tested the minimum difference in *Q *we could detect by constraining the extent to which the two *Q*s could vary from one another on 8- and 16-taxon double-*Q *phylogenies (Figure 6).

**Figure 9:**
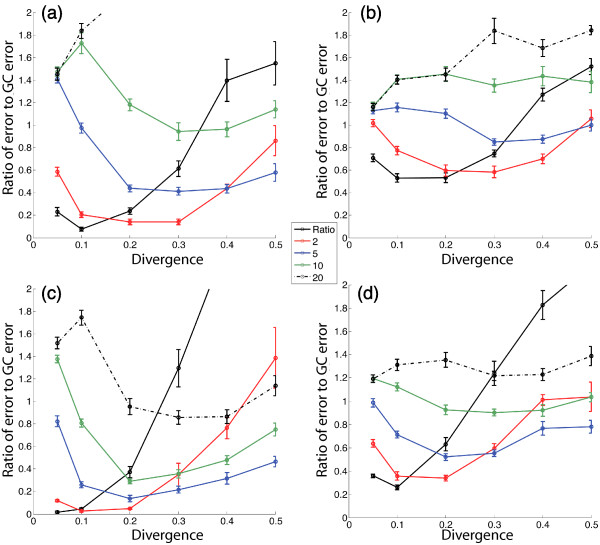
Performance of our new statistics compared to the performance of GC content. Each data point corresponds to a sample of independent simulations. We determine the error rate (as shown in Figure 8) both for GC content and for our statistic 36. Here we show the error rate for statistic 36 divided by the error rate for GC-content alone. When the ratio of error rates is less than 1, our new statistic outperforms GC content in detecting simulated HGT events. The different panels show different simulation conditions: (a) 8-taxon trees, no restriction on *Q*_2_, (b) 8-taxon trees, each random element of *Q*_2 _constrained to be within 90% of the corresponding element of *Q*_1_, (c) 16-taxon trees, no restriction on *Q*_2_, and (d) 16-taxon trees, each random element of *Q*_2 _constrained to be within 90% of the corresponding element of *Q*_1_. Different curves correspond to different branch ratios. Error bars represent the standard error of the mean for 10 independent simulations. This statistic outperforms GC content substantially when the branch ratio is low (corresponding to a more ancient split of the host species from the original common ancestor) and the divergence is low to intermediate (gene sequences have intermediate to high sequence identity). In some cases we find that use of this statistic gives a lower error rate than does GC content by a factor of 10.

Some of our new statistics outperformed GC-content substantially when used as single-variable classifiers. For example, using 16-taxon trees with divergence = 0.1 and a branch ratio of 1 or 2, statistic 41 gave a 10-fold decrease in error rate relative to GC content. The improvement was largest when the changes in the rate matrix were unrestricted (i.e. the two rate matrices were chosen at random, rather than being constrained to be within some percentage of each other), and when the branch ratio of internal to external branches was low (set at 1 or 2). This result was expected because long internal branches lead to very different sequence compositions, which are easily identifiable by GC content changes alone. Several of our new statistics consistently outperformed GC content when both the overall divergence and the ratio of divergence in the inner and outer branches were relatively low (Figure 9). These conditions model cases under which HGT might be mistaken for differential loss of members of a gene family. Our ability to detect HGT more sensitively under these conditions is thus encouraging.

### Combinations of statistics reduce the error rate by an order of magnitude

To assess the performance of different statistics, both alone and in combination, over the entire data set, we used the random forests algorithm [75]. Random forests allow us to determine classifiers that combine multiple statistics and to identify the combinations of statistics that gave most weight to the optimal classifiers (see Table 1 for a description of each statistic). We found that no single statistic performed well under all circumstances. The best performer overall, statistic 33, accounted for only 7.7% of the variable importance across all classifiers, and was closely followed by statistic 28 (6.9%) and statistic 32 (6.8%).

These three statistics with highest variable importance are mathematically different and uncorrelated. Although all use three-sequence comparisons rather than pairwise comparisons, 33 and 28 use SVD on the scaled and unsealed Q^
 MathType@MTEF@5@5@+=feaafiart1ev1aaatCvAUfKttLearuWrP9MDH5MBPbIqV92AaeXatLxBI9gBaebbnrfifHhDYfgasaacH8akY=wiFfYdH8Gipec8Eeeu0xXdbba9frFj0=OqFfea0dXdd9vqai=hGuQ8kuc9pgc9s8qqaq=dirpe0xb9q8qiLsFr0=vr0=vr0dc8meaabaqaciaacaGaaeqabaqabeGadaaakeaacuWGrbqugaqcaaaa@2DE7@*t*_*i *_respectively, and 32 uses the mean distance of each Q^
 MathType@MTEF@5@5@+=feaafiart1ev1aaatCvAUfKttLearuWrP9MDH5MBPbIqV92AaeXatLxBI9gBaebbnrfifHhDYfgasaacH8akY=wiFfYdH8Gipec8Eeeu0xXdbba9frFj0=OqFfea0dXdd9vqai=hGuQ8kuc9pgc9s8qqaq=dirpe0xb9q8qiLsFr0=vr0=vr0dc8meaabaqaciaacaGaaeqabaqabeGadaaakeaacuWGrbqugaqcaaaa@2DE7@*t*_*i *_from the mean. The three best statistics were all based on USVD, although both USVD and CSVD-based statistics contributed to the best ten and together provide highly sensitive discrimination. In general, the best statistics were not highly correlated with one another. Pairwise correlation coefficients among the best 10 statistics averaged *r *= -0.001, ranging from -0.38 to 0.68.

Of the 45 statistics we tested, GC content (statistic 45) placed llth. This relatively poor showing underscores the importance of using the full rate matrix, rather than simply examining the composition. However, GC content does work well when the sequences have diverged markedly in composition, especially when the internal branches are very long (i.e., the ratio between internal and terminal branches is high).

We also found that using three-sequence estimates of the rate matrix (which allows non-time-reversible changes to be estimated) provided far more power to detect changes in *Q *than did pairwise estimates (which can only detect time-reversible changes). Of the top 10 statistics in the combined classifier, all were based on three-sequence comparisons (average variable importance 5.1%), whereas 9 of the 10 worst statistics were based on pairwise comparisons (average variable importance 0.51%). This difference indicates that three-sequence estimates of *Q *perform significantly better than pairwise estimates. Thus, eliminating the assumption of time-reversibility is important for detecting HGT.

**Figure 10:**
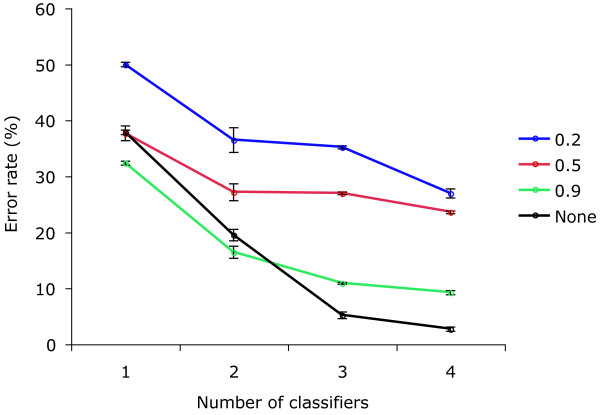
Classification results when multiple statistics are combined. The error rate decreases as more variables are used for classification. Results are averaged across branch length, number of taxa, and ratio of terminal to internal branches. Each simulation was weighted equally. Different curves correspond to different restrictions on the rate matrices used in the simulation (see Figure 6). In the "unconstrained" case, the second rate matrix *Q*_2 _was chosen randomly, independent of the original *Q*_1_. In the "constrained" case, each random element of *Q*_2 _must be within 20%, 50%, or 90% of the corresponding element of *Q*_1_. Adding variables decreased the error rate in all cases, decreasing the error rate from 50% to 27% when the rate matrix change is 20% per element and from 38% to 2.7% when the change in *Q *is unrestricted. The statistics added were, in order of use, statistics 37, 32, 36, and 34.

We started with the single best statistic and added back each additional statistic (Figure 10) to test how using combinations of statistics improves the accuracy of classification. We found that adding statistics dramatically decreases the error rate for a wide range of parameter settings. This effect is most dramatic when changes in the rate matrix are unrestricted: going from the single best statistic to the combination of the four best statistics reduces the error rate more than 10-fold, from 38% to 2.7%. The corresponding error rate for GC content alone with the same data was 39% (i.e. GC content performed almost as well as the best single classifier on this data set, but performed much more poorly on the combined classifier). These figures reflect the performance of a trained classifier on new data, so are unlikely to be vulnerable to overfitting.

## Discussion and conclusions

In this paper, we developed new measures which use sequence data to predict whether or not a gene has been horizontally transferred. These measures are based on apparent changes in the micleotide substitution dynamics that occur when a gene is transferred to a new organism; these changes can be described by changes in the micleotide substitution rate matrix *Q*. We used simulated sequence evolution to assess our ability to infer the rate matrix and use changes in the rate matrix to assess horizontal transfer. Our results show that we are able to accurately infer the rate matrix from simulated sequence data. Using a combination of statistics derived from SVD of a set of rate matrices inferred from different pairs or triples of genes within a single phylogenetic tree, simulated HGT events can be detected with an error rate as low as 2.7%. Our new measures of horizontal transfer demonstrate greatly improved accuracy and sensitivity in the detection of simulated horizontal transfer: using a four of our new statistics gives a factor of 19 decrease in the error rate of simulated HGT detection, relative to the error rate of HGT detection solely by changes in GC content. Thus, we have demonstrated a powerful new class of methods for detecting HGT that combine some of the best features of existing phylogenetic and compositional methods.

Our results demonstrate that taking the micleotide substitution rate matrix into account provides substantial advances over simply examining the composition of the sequences. In addition, we show that the combination of different SVD-based statistics is an efficient way of making use of these data. As we expected, no single statistic performs well for all simulation conditions. This result, especially the different performance between methods at different levels of sequence divergence (Figure 9), is consistent with previous observations that different composition-based statistics may be most effective for changes that occurred at different times during evolutionary history [19].

Many changes in the rate matrix would be expected to leave GC content unchanged. The micleotide substitution rate matrix can thus be thought of as generalizing the concept of GC content to take into account the dynamics of compositional change as well as the composition of modern genes. Therefore, as our results show, examination of changes in the rate matrix can be more powerful than examination of composition alone.

We were particularly interested in understanding whether the assumption of a time-reversible Markov process affects the ability to detect HGT from sequence data. We found that using methods that do not assume time reversibility is critical to detecting HGT in our simulated phylogenies.

In many ways, our simulations were more restrictive than the conditions that exist in real sequences. For example, our phylogenies simulate temporary changes in *Q *in the past, rather than trees in which *Q *changes and in which the new *Q *persists to the present. The temporary change in the rate matrix leaves a weaker signature in the sequence than does a persistent change. We require that genes share a common ancestor that has evolved for part, but not all, of its history under a different *Q *from the modern species in which it is found. Detecting these transient changes in *Q *is more difficult than simply testing whether *Q *has changed anywhere in the phylogenetic tree [43]. Sequences that currently share a genome experience mutational processes that reflect the same *Q*, blurring distinctions that may have accumulated in the past when the sequences were found in different genomes.

Our results on both simulated and real sequences show that our method is likely to identify short blocks of ten or more transferred genes, including difficult-to-detect cases in which genes replace orthologs already present in the genome. Large errors in the inference of *Q *from single genes, largely due to sampling error, limit our ability to observe the transfer of short fragments of DNA. However, many HGT events involve multiple genes: a recent study of genomic islands showed that a sample of 89 transferred blocks reported in the literature contained 41 genes on average [76]. We thus expect the new methods to be useful for uncovering a wide range of transfer events, especially those mediated by plasmids or phages [5].

The analysis of bacterial sequence data demonstrates that we can detect changes in the rate matrix even between closely related genomes with similar GC content, including different strains of the same species. This further confirms our hypothesis that the use of the full rate matrix can detect differences in sequence divergence with greater power than compositional statistics. We thus expect that this technique will be useful (*i*) for detecting recent transfers across large taxonomic groups (e.g. across divisions) when both donor and recipient genomes have similar overall composition, and (*ii*) for detecting transfers of blocks of genes between closely related genomes. This latter case of orthologous gene displacement is thought to be important in the transfer of antibiotic resistance (e.g. [27]).

Our improved ability to detect ancient changes in *Q *will be especially important for detecting subtle changes in biological sequences that compositional and phylogenetic methods alone cannot resolve. Our ability to reliably detect per-element changes in *Q *as small as a factor of two will be especially important for resolving transfers between species with similar *Q*. This may assist in the detection of transfers between closely related lineages, which often have similar *Q*. To our knowledge, this work is the first time that sensitivity of any method, including GC-content, to variations in the rate matrix has been measured. We expect that our new methods will greatly enhance our ability to detect horizontal gene transfer and a wide range of other alternative phylogenetic events throughout the tree of life.

## Methods

### Sequence simulations

Sequences were simulated by a Monte Carlo procedure (Figure 5). First, we define a phylogenetic tree topology and branch lengths, where branch lengths represent time and branch points represent species divergence events. Then we specify the nucleotide substitution rate matrix as a function of time, either by applying a single *Q *to the whole phylogeny or specifying *Q *at each node. (When a rate matrix is specified at a given node, it applies to the downstream branch of the tree, until the next node.) We then calculate *P *at each node by exponentiating the product of *Q *and the upstream branch length. Finally, we set a random sequence at the root and use the *P *at each node to mutate the sequence of its parent. Simulations shown here were for phylogenies of 8 or 16 sequences (taxa), starting with an input sequence of equal nucleotide frequencies, with sequence lengths of 1000 nucleotides evolving on the DNA alphabet (T, C, A, G). For sensitivity analysis, we set all branch lengths equal except for the branch length from the root to the first internal nodes. The lengths of these branches that started at the root were scaled by the same constant factor, which we called the branch ratio. This difference in branch length for the internal nodes allowed us to test the effect of the relative importance of changes in *Q *in different parts of the phylogenetic tree.

HGT phylogenies were generated by changing *Q *along one internal branch of the tree. This procedure simulates a period of evolution in a lineage with a different rate matrix, followed by transfer back to a lineage with the original rate matrix. See Figure 6 for a detailed explanation of each parameter varied in these simulations.

### Inference of nucleotide substitution rate matrices

Each nucleotide substitution rate matrix *Q *was inferred by empirically deriving P^
 MathType@MTEF@5@5@+=feaafiart1ev1aaatCvAUfKttLearuWrP9MDH5MBPbIqV92AaeXatLxBI9gBaebbnrfifHhDYfgasaacH8akY=wiFfYdH8Gipec8Eeeu0xXdbba9frFj0=OqFfea0dXdd9vqai=hGuQ8kuc9pgc9s8qqaq=dirpe0xb9q8qiLsFr0=vr0=vr0dc8meaabaqaciaacaGaaeqabaqabeGadaaakeaacuWGqbaugaqcaaaa@2DE5@ from a pair or rooted triple of sequences (using the most divergent sequence as the outgroup), and obtaining Q^
 MathType@MTEF@5@5@+=feaafiart1ev1aaatCvAUfKttLearuWrP9MDH5MBPbIqV92AaeXatLxBI9gBaebbnrfifHhDYfgasaacH8akY=wiFfYdH8Gipec8Eeeu0xXdbba9frFj0=OqFfea0dXdd9vqai=hGuQ8kuc9pgc9s8qqaq=dirpe0xb9q8qiLsFr0=vr0=vr0dc8meaabaqaciaacaGaaeqabaqabeGadaaakeaacuWGrbqugaqcaaaa@2DE7@*t *by taking the matrix logarithm of P^
 MathType@MTEF@5@5@+=feaafiart1ev1aaatCvAUfKttLearuWrP9MDH5MBPbIqV92AaeXatLxBI9gBaebbnrfifHhDYfgasaacH8akY=wiFfYdH8Gipec8Eeeu0xXdbba9frFj0=OqFfea0dXdd9vqai=hGuQ8kuc9pgc9s8qqaq=dirpe0xb9q8qiLsFr0=vr0=vr0dc8meaabaqaciaacaGaaeqabaqabeGadaaakeaacuWGqbaugaqcaaaa@2DE5@. Each *Q *was initially estimated as Q^
 MathType@MTEF@5@5@+=feaafiart1ev1aaatCvAUfKttLearuWrP9MDH5MBPbIqV92AaeXatLxBI9gBaebbnrfifHhDYfgasaacH8akY=wiFfYdH8Gipec8Eeeu0xXdbba9frFj0=OqFfea0dXdd9vqai=hGuQ8kuc9pgc9s8qqaq=dirpe0xb9q8qiLsFr0=vr0=vr0dc8meaabaqaciaacaGaaeqabaqabeGadaaakeaacuWGrbqugaqcaaaa@2DE7@ = ln P^
 MathType@MTEF@5@5@+=feaafiart1ev1aaatCvAUfKttLearuWrP9MDH5MBPbIqV92AaeXatLxBI9gBaebbnrfifHhDYfgasaacH8akY=wiFfYdH8Gipec8Eeeu0xXdbba9frFj0=OqFfea0dXdd9vqai=hGuQ8kuc9pgc9s8qqaq=dirpe0xb9q8qiLsFr0=vr0=vr0dc8meaabaqaciaacaGaaeqabaqabeGadaaakeaacuWGqbaugaqcaaaa@2DE5@. As shown in Figure 3, the error in Q^
 MathType@MTEF@5@5@+=feaafiart1ev1aaatCvAUfKttLearuWrP9MDH5MBPbIqV92AaeXatLxBI9gBaebbnrfifHhDYfgasaacH8akY=wiFfYdH8Gipec8Eeeu0xXdbba9frFj0=OqFfea0dXdd9vqai=hGuQ8kuc9pgc9s8qqaq=dirpe0xb9q8qiLsFr0=vr0=vr0dc8meaabaqaciaacaGaaeqabaqabeGadaaakeaacuWGrbqugaqcaaaa@2DE7@ is comparable to the intrinsic sampling error that is inevitable with sequences of finite length.

Figure 5 contains results of a typical run: we show the ancestral sequence and rate matrix, the evolved sequences, and the inferred rate matrix. Our results, which we obtained without using model parameters such as *Q *and the ancestral sequences, demonstrate that we will be able to recover accurate rate matrices from modern biological sequences if they evolve under the model that we assume.

### Inferring change within a set of rate matrices

We used several strategies for combining a set of rate matrices from a single phylogenetic tree into a single statistic indicative of HGT (Table 1). These methods used both two- and three-sequence comparisons to test whether the assumption of a time-reversible model affected our ability to detect HGT. (Note that a model inferred from two-sequence comparisons must be time reversible, because one cannot determine which character is older from the fact that a change occurs. In three-sequence comparisons, a direction of change can be inferred.) Although most of the statistics were based on centered or uncentered SVD (see Theory), some were based on the mean or variance of the distance between each individual rate matrix and the mean of all inferred rate matrices. We also used three different strategies for combining the eigenvalues of the set of *Q*_*i*_: the ratio of the first and second eigenvalues, the ratio of the first eigenvalue to the sum of all eigenvalues, and the statistic introduced by Weiss et al. [43]. This last statistic is ∑_*i *_ln (1 + *σ*_*i*_), where *σ*_*i *_is the *i*th eigenvalue.

### Measuring variation in the rate matrices within and between species

We selected fully sequenced bacterial genomes (*i*) to test whether rate matrices vary more between species than within them, and (*ii*) to determine how many genes are required in practice for reliable inference of the rate matrix. We selected sets of three species ('triples') for comparison. Our selection criteria were (a) the third codon position divergence (for genes that are present in all three species) between the two sister species was between 5% and 15% on average, (b) the third codon position divergence between each of the two sister species and the outgroup was between 10% and 25% on average, and (c) the mode of the distribution of divergences for individual genes was within 30% of the mean. The third criterion was important because of the mosaic structure of certain genomes, which may have been subject to the integration of large plasmids (e.g. the Locus for Enterocyte Effacement (LEE) plasmid required for virulence in enter opathogenic *E. coli *has apparently integrated into the *Citrobacter rodentium *genome [77]) or which may have extreme compositional bias between the two strands (e.g. Borrelia burgdorferi [46]).

After choosing appropriate sets of species, we applied additional selection criteria for individual genes. We considered only genes (a) that were present in the KEGG Orthology (KO) [78] in all three species within a given triple, (b) that were not identical between any pair of species, (c) that aligned over a span of at least 100 amino acids, and (c) in which the rank order of the divergences at each codon position was third > first > second, reflecting known constraints on coding sequences [68,69]. We also excluded genes that were (d) less than 5% divergent or (e) more than 20% divergent at the third codon position between the sister groups. These constraints eliminated from consideration misaligned sequences and sequences that were likely to have been horizontally transferred, and sequences with too few changes to allow accurate inference of the rate matrix.

Using these selection criteria, we identified ten representative genomes for rate matrix analysis. The genome codes from KEGG for these triples were BUR (*Burkholderia sp. 383*) and BPM (*Burkholderia pseudomallei 1710b*), using BPA (*Bordetella parapertussis*) as an outgroup; BTE (*Burkholderia thai-landensis *and BPS (*Burkholderia pseudomallei K96243*), using TBD (*Thiobacillus denitrificans*) as an outgroup; PSP (*Pseudomonas syringae pv. phaseolicola 1448A*) and PSB (*Pseudomonas syringae pv. syringae B728a*), using PFL (*Pseudomonas fluorescens Pf-5*) as an outgroup; SMA (*Streptomyces avermitilis*) and SCO (*Streptomyces coelicolor*), using NFA (*Nocardia farcinica*)*as *an outgroup, and PFO (*Pseudomonas fluorescens PfO-1*) and PFL (using PSB as an outgroup). These species spanned three bacterial divisions (beta proteobacteria, gamma proteobacteria, and actinobacteria). They were also relatively consistent in GC content, ranging from 59.0% for PFO to 71.4% for SCO. All pairs of sister taxa were within 1% GC content overall: for comparison, the standard deviation of GC content for genes within a species ranged from 2.6% for PFO to 4.0% for BPS.

We aligned the protein sequences for the genes that met our selection criteria using MUSCLE [79], because amino acid alignments are more reliable than nucleotide alignments for divergent sequences [80]. We then threaded the nucleotide sequences back through the amino acid alignment, extracted the parts of the aligned sequences corresponding to third codon positions, and chose 100 samples of genes at random from each triple. In each of the 100 samples, we varied the number of genes *n *from 1 to 50. We inferred the rate matrix for each sample using the methods described above for simulated sequences (summing the counts within each sample, converting these counts to probabilities, taking the log of the probability matrix, and normalizing the trace to 1). We then measured the Euclidean distance between the rate matrices of pairs of samples. We expected that within-genome comparisons, in which both samples of genes came from the same genome, would yield pairs of rate matrices with smaller mean distances than would between-genome comparisons, in which the two samples came from different genomes.

### Development of optimal classifiers

We identified optimal classifiers empirically using the standard procedure for dividing a set of observations into two classes in a way that minimizes the error rate [74]. First, we pooled the values of each statistic for both HGT and non-HGT phylogenies into a list sorted by the value of the statistic. Second, for each value of the statistic, we counted the number of errors as the minimum of (a) the sum of non-HGT values greater than that value and HGT values less than that value, or (b) the sum of HGT values greater than that value and non-HGT values less than that value. The optimal classifier is the boundary that gives the minimum error rate. For example, suppose 5 of 100 HGT phylogenies had a value of a statistic of less than 12, and 10 of 100 non-HGT phylogenies had a value of the same statistic of greater than 12. A dividing line drawn at 12 would result in two categories: one with 5 HGT trees and 90 non-HGT trees, and the other with 95 HGT trees and 10 non-HGT trees. The number of errors would thus be either (10 + 5) and (90 + 95), depending on whether we call the trees above or below the boundary the HGT trees. Taking the minimum of these two values, we have an error rate of 15 out of 200, or 7.5%.

To test which of these statistics had the greatest effect on our ability to discriminate HGT phylogenetic trees from non-HGT phylogenetic trees, and to minimize issues with overfitting in the procedure above, we also used the random forests classification method (Salford Systems, University of California, Berkeley) [75]. Random forests is a supervised learning algorithm that extends methods based on classification trees. A classification tree defines a hierarchy of decisions based on features of the data, at the end of which an observation is assigned to one of a defined number of categories [81] (in this case, HGT or non-HGT). Instead of building a single classification tree, the random forests algorithm builds a set of random classification trees (a forest) that classifies objects based on an input vector of properties. In our case, the two classes are phylogenies either containing or not containing an HGT event, and the vector consists of the 45 statistics we measured for each phylogeny (Table 1). Each classification tree classifies the phylogenies using a random subset of the data. The classification trees that are most successful at voting for the correct classifications on a training set (i.e. inclusion decreases the error rate at which HGT phylogenies are classified as non-HGT, or vice versa) are kept while the unsuccessful classification trees are discarded. After many iterations, the forest becomes highly successful at classifying the different phylogenies. The advantages of random forests over other classifiers are that it provides a consistently lower error rate and is less prone to overfitting than techniques such as neural networks, SVMs, or Bayesian belief networks [75], and it directly estimates which statistics are important for the classifications.

We built 500 trees per forest with 14 predictors per node. The number of predictors was approximately twice the square root of the number of classifiers, as recommended for optimal results [75] (we used 45 classifiers in the analysis). A technique called out-of-bag error estimate was used for protection from overfitting [75].

Random forests provides information about the importance of each variable (statistic) in the classifier. The importance of each variable is expressed as a percentage of that statistic's contribution to the overall optimal classifier (Table 1). Thus, a statistic with high variable importance accounts for more of the accuracy in assigning trees to the HGT or non-HGT categories.

## Authors' contributions

M. H. performed statistical analyses. M. B. and R. K. together developed the mathematical methods. R. K. implemented the mathematical methods and designed the simulations. All three authors contributed to the manuscript.
